# Paeoniflorin Inhibits the Proliferation and Metastasis of Ulcerative Colitis-Associated Colon Cancer by Targeting EGFL7

**DOI:** 10.1155/2022/7498771

**Published:** 2022-08-28

**Authors:** Yunxia Wang, Yi Zhou, Hui Lin, Haiyan Chen, Shu Wang

**Affiliations:** ^1^Department of Surgery, Shidong Hospital of Shanghai Yangpu District, Shanghai 200438, China; ^2^Department of Anesthesiology, Shidong Hospital of Shanghai Yangpu District, Shanghai 200438, China; ^3^Urology Surgery, Shidong Hospital of Shanghai Yangpu District, Shanghai 200438, China

## Abstract

In this study, we studied the therapeutic potential of PF in mouse models of CAC. PF could inhibit the proliferation, migration, invasion, and clone formation and promote the apoptosis of colon cancer cells. Furthermore, PF showed a good effect on inhibiting the aggregation and infiltration of inflammatory cells, protecting the intestinal mucosal barrier, and inhibiting the growth of colon tumors in the AOM/DSS-induced CAC model. PF also significantly inhibited the expression of TNF-*α*, IL-1*β*, IL-6, IL-13, and CEA-related inflammatory factors in the AOM/DSS-induced CAC model by inhibiting the TLR4/NF-*κ*B signaling pathway and EGFL7 expression. Therefore, PF showed a potential therapeutic effect on mice in the CAC model by inhibitingTLR4/NF-*κ*B mediated inflammatory response and EGFL7 expression.

## 1. Introduction

Inflammatory bowel disease (IBD) is a disease affecting millions of people, including ulcerative colitis (UC) and Crohn's disease (CD) [1]. UC is a chronic inflammatory bowel disease with unknown etiology. Its pathogenesis is related to environmental, genetic, immune, and other factors. Ulcerative colitis can lead to the destruction of the intestinal epithelial mucosal barrier, the activation of immune cells, and the production of a large amount of TNF-*ɑ*, IFN-*γ*, IL-6, and other proinflammatory cytokines and induce epithelial cell apoptosis. IBD is a key risk factor for colitis-associated cancer (CAC). However, existing drugs for treating IBD and preventing CAC do not work well or have serious side effects. New and improved drugs are necessary. Therefore, there is an urgent clinical need for newer and more improved safe and effective drugs.

Paeoniflorin (PF) is the main component of Chinese medicine Paeoniae Radix Alba and Paeoniae Rubra which is first isolated in 1963. Paeoniflorin has the advantages of anti-inflammatory, heat removal, antispasmodic, nerve and brain protection, antimyocardial ischemia, clearing free radicals in the body, immune regulation, and other pharmacological effects [2, 3]. It has been shown that PF can inhibit nitric oxide, TNF-*ɑ*, and IL-6 production in LPS-stimulated RAW264 cells [4]. Furthermore, it has also been shown that PF can reduce TNF-*ɑ*, IL-6, and IL-1*β* levels by inhibiting NLRP3 inflammatory signaling, thus protecting against inflammatory pain [5]. Furthermore, PF can ameliorate cholestasis and protect the liver transporters by inhibiting the NF-kB and IL-1*β*-associated inflammation [6]. However, there are few studies on whether PF can treat UC and CAC and the associated mechanisms of action.

Epidermal growth factor-like domain (EGFL7) is a newly discovered secretory protein in recent years. Some studies have proved that it plays an important role in the tube formation process of embryonic vascular development. Recent studies have found that it can significantly enhance the movement and migration ability of endothelial cells and fibroblasts, suggesting that it has the biological function of regulating cell movement and migration [7, 8]. However, EGFL7 has not been reported in the invasion and metastasis of colon cancer cells. This study found that PF was able to relieve the symptoms in UC and CAC model mice by inhibiting the EGFL7 expression.

This study intends to analyze the role of EGFL7 in inflammatory bowel cancer through in vivo and in vitro experiments. We also analyzed whether PF could inhibit the proliferation and metastasis of ulcerative colitis-associated colon cancer by targeting EGFL7.

## 2. Materials and Methods

### 2.1. Colon Cancer Tissue Collection

The colon cancer tissues of 15 patients were provided by Shidong Hospital of Shanghai Yangpu district. There were 10 males and 5 females with an age range of 39–78 years, with an average age of 57.2 ± 12.9 years. Colorectal cancer tissue and corresponding paracancer tissue 5 cm from the edge of colorectal cancer tissue were collected from each patient. Tissue specimens after radical resection of colorectal cancer were confirmed by two pathologists. None of the patients received preoperative chemotherapy or radiotherapy. This study was approved by the Medical Ethics Committee of Shidong Hospital of Shanghai Yangpu district. Informed consent was signed by all patients and their families.

### 2.2. Establishment of Animal Models of Colitis-Associated Cancer (CAC)

Female C57BL/6 mice (18–22 g) were purchased from the Animal Center of the Academy of Military Medical Sciences (Beijing, China), 6 in each group. They were fed in a sterile environment and adapted to the environment for 7 days before the experiment. 30 BALB/c mice were randomly divided into the control group (control), model group (AOM), and paeonionin group (PF). The establishment of CAC mouse models can be performed with reference to publicly available data [9]. AOM was dissolved in saline and prepared in solution form. BALB/c mice were intraperitoneally injected with 12 mg/kg AOM solution every day for the first five days, and then BALB/c mice were provided with drinking water containing 2% DSS for seven consecutive days and entered the recovery period of 14 days. The above process is a complete induction cycle, which is repeated three times to establish the CAC animal model. The mice in the control group were only treated with saline. At the beginning of the third cycle, mice in the model group began to take paeoniflorin (3 g/kg) until the end of the experiment. After the mice were dissected, the number of tumors on the colonic tissue was counted, the tumor volume was measured, and then part of the colonic tissue was preserved in formalin solution for subsequent experiments.

### 2.3. Hematoxylin and Eosin (HE) Staining Experiment

The colonic tissue was removed from the formalin solution and rinsed with running water to remove formalin. The colonic tissue was trimmed and dehydrated overnight in a dehydrator and embedded in paraffin. The embedded tissue was sliced and placed on a slide. After the slices were treated in the oven for some time, they were dewaxed with different concentrations of toluene and treated with different concentrations of ethanol and finally stained with eosin and hematoxylin. The morphological changes of colon tissue in different groups were observed under a microscope.

### 2.4. Immunohistochemical (IHC) Experiment

The colon tissue sections were treated with different concentrations of toluene and ethanol and then washed with ddH_2_O. Then, sodium citrate buffer solution was added, and antigen repair was carried out in a microwave oven, that is, the closed antigen sites were exposed. The slices were placed in a 3% hydrogen peroxide solution for 15 minutes and then washed sequentially with ddH_2_O and PBS buffer. The residual PBS solution on the slide was dried, and then normal goat serum was added to the colonic tissue and placed at room temperature for 20 minutes. Next, prediluted primary antibody, EGFL7 human antibody (ab256451,1 : 500, Abcam), was added to the tissue overnight at 4°C. The primary antibodies on the tissues were washed with PBS and then treated with horseradish peroxidase-labeled secondary antibodies for 2 h at room temperature. After washing the slides with PBS, tissue staining was performed with DAB (ab64238, Abcam), and hematoxylin was used for nuclear staining. Finally, the tissue was dehydrated in different concentrations of ethanol and xylene.

### 2.5. Real-Time PCR Test

Colonic tissue was first treated with TRIZOL and chloroform and then centrifuged for 15 min at 12000 rpm. The supernatant was retained and treated with isopropanol for 5 min and then centrifuged again in the centrifuge for 10 min at 12,000 rpm. The supernatant was discarded, and 75% ethanol solution was added followed by centrifuging for 10 minutes at 12,000 rpm. All of the above steps are operated on ice or at low temperatures. Finally, the upper ethanol solution was discarded, the RNA was dissolved in enzyme-free water, and its concentration was determined.

The RNA was predenatured and denatured at 94°C, annealed at 57°C and extended at 72°C, and was repeated for a total of 30 cycles. Among these, the last cycle was performed at 72°C and the RNA extension operation for 10 min. The content of the relevant mRNA was then measured separately. The 2^−ΔΔCt^ method was used for quantitative PCR calculation.

### 2.6. Cell Culture, EGFL7 Silencing Expression, and PF Treatment

Normal colonic epithelial cells HIEC-6(CRL-3266) were purchased from ATCC. And, colorectal cancer cells HCT-116(CCL-247), HCT-8(CCL-244), SW620(CCL-227), LOVO(CCL-229), and SW480(CCL-228) were purchased from ATCC. HIEC-6, LOVO, and SW480 cells were cultured with a DMEM medium containing 10% FBS. HCT-116, HCT-8, and SW620 were cultured with RPMI1640 medium containing 10% fetal bovine serum (FBS). All cells were cultured at 37°C, with 5% CO_2_.

The LOVO and SW480 cells were divided into negative control groups (sh-NC) and experimental groups (sh-EGFL7). The negative control group was only transfected with vector, and the experimental group was transfected with EGFL7-siR. Before transfection, the cells were sealed in 6-well plates to ensure that the cell density was about 70% for transfection. In the experimental group, EGFL7-siR was transfected into LOVO and SW480 cells using Lipofectamine 3000 (Invitrogen, USA). After 48 hours, the SW480 cells were treated with different PF concentrations (40 *μ*g/ml, 80 *μ*g/ml, and 160 *μ*g/ml) for 24 hours.

### 2.7. CCK8 Cell Proliferation Detection

LOVO and SW480 cells were seeded in 96-well plates and routinely cultured in a 37°C, 5%CO_2_ cell culture incubator for 24 h. Then, the cell culture medium was replaced with a fresh DMEM medium containing 40 *μ*g/ml of PF. After further culturing for 24 h, 48 h, and 72 h, respectively, CCK-8 (ab228554, Abcam) reagent was added and continued to culture for 4h. Finally, the absorbance values were detected at 480 nm.

### 2.8. Cell Scratch Assay

Use the marker pen to draw a horizontal line evenly on the back of the 6-well plate. The LOVO and SW480 cells were then seeded in a 6-well plate and scraped the cell monolayer in a straight line to create a “scratch” with a pipet tip. Remove the debris and smooth the edge of the scratch by washing the cells with PBS and cultured in serum-free DMEM medium in the 37°C incubators. Cells were observed under a microscope and photographed at 0 h, 6 h, 12 h, and 24 h, respectively. Finally, the distance between the cell was calculated using the ImageJ software.

### 2.9. Cell Invasion Experiment

The matrix gel was dissolved overnight in a refrigerator at 4°C, and the next day, the matrix gel was diluted in the ratio of 1: 8 with the culture medium. 40 *μ*L of matrix gel was added to transwell upper chamber and placed in the 37°C incubators for 30 min. LOVO and SW480 cells were suspended in serum-free medium, and 100 *μ*L cell suspension was added into the upper chamber, and 500 *μ*L DMEM medium containing 20% fetal bovine serum was added into the lower chamber. The 24-well plates containing the transwell chamber were incubated in a 37°C incubator for 48 h. The cells were washed three times with PBS and fixed with 4% paraformaldehyde for 30 min. Subsequently, the cells were stained with 0.1% crystal violet for 30 min, and then noninvasive tumor cells and matrix gel were removed with a cotton swab. Five visual fields were randomly selected under the inverted microscope to count the number of invasive tumor cells.

### 2.10. Cell Clone Formation Assay

LOVO and SW480 cell suspensions were prepared and diluted with the medium. The dishes containing cells were placed in the 37°Cincubator for 2 to 3 weeks until the clone was formed. The medium was discarded, and the cells were fixed with 4% paraformaldehyde for about 15 min. After the paraformaldehyde solution was discarded, an appropriate amount of GIMSA staining solution was added to the dishes for 20 min. The number of cell clones formed was counted under a microscope.

### 2.11. TUNEL Assay

A one-step TUNEL apoptosis detection kit (Beyotime, Shanghai, China) was used to estimate the apoptosis efficiency according to the manufacturer's instructions.

### 2.12. Statistical Analysis

Data were analyzed using SPSS23.0 software, all data were expressed as mean standard deviation, and differences between groups were compared by ANOVA. *P* < 0.05 represented statistically significant differences.

## 3. Results

### 3.1. EGFL7 Is Upregulated in Ulcerative Colitis-Associated Colon Cancer

**Figure 1:**
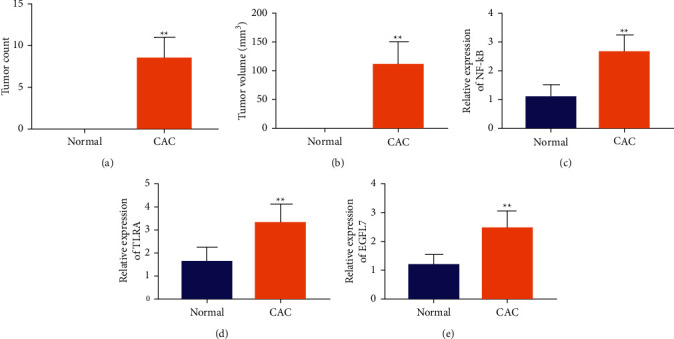
EGFL7 is upregulated in ulcerative colitis-associated colon cancer. (a, b) The number and volume of tumors of mice. (c–e) RT-qPCR was used to test the expression of NF-*κ*B, TLR4, and EGFL7 in the colon tissue of CAC mice. ^*∗∗*^*p* < 0.01.

We established the CAC mouse model with AOM/DSS and further detected the expression of NF-*κ*B, TLR4, and EGFL7. Compared with the normal control group, many tumors appeared on the colon tissue of mice in the CAC model group. Figures 1(a) and 1(b) show the number and volume of tumors on the colon tissue of mice in the CAC model, respectively. Moreover, the RT-qPCR results showed that the expression of NF-*κ*B, TLR4, and EGFL7 increased significantly in the colon tissue of CAC mice (Figures 1(c)–1(e)). The above experimental results show that there are a large number of inflammatory aggregates in the colonic tissue of CAC mice, intestinal mucosal tissue is damaged, and even a lot of tumors in the colonic tissue. Moreover, the expression of EGFL7 related protein in colon tissue of mice with CAC is increased.

### 3.2. Overexpression of EGFL7 in Colon Cancer

**Figure 2:**
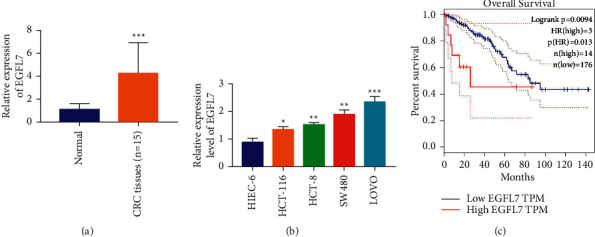
Overexpression of EGFL7 in colon cancer. (a) RT-qPCR experiments was used to detect the mRNA content of EGFL7 in colon cancer patients. (b) RT-qPCR experiment was used to detect the mRNA content of EGFL7 in colon cancer cells and normal colon cells. (c) The overall survival of colon cancer patients with different EGFL7 expression levels. ^*∗*^*p* < 0.05, ^*∗∗*^*p* < 0.01, and ^*∗∗∗*^*p* < 0.001.

To further study the expression of EGFL7 in colon tissue of normal and colon cancer patients, IHC and RT-qPCR experiments were carried out, respectively, and the relationship between EGFL7 expression and patient overall survival was analyzed. Moreover, the results of the RT-qPCR showed that the mRNA content of EGFL7 was significantly higher in colon cancer patients than in normal colonic tissue (Figure 2(a)). We compared the expression level of EGFL7 between different colon cancer cells and normal colon cells by RT-qPCR. The results showed that EGFL7 expression was significantly higher in colon cancer cells than in normal colon cells HIEC-6, wherein the EGFL7 expression in colon cancer cells ranging from low to high was HCT-116, HCT-8, LOVO, and SW480, respectively (Figure 2(b)). Since EGFL7 has the highest expression in LOVO and SW480, these two cell lines were selected for subsequent studies. We also analyzed the overall survival of colon cancer patients with different EGFL7 expression levels. The results showed that patients with high EGFL7 expression had significantly lower survival and poor prognosis(Figure 2(c)). The above in vivo and in vitro results show that EGFL7 is highly expressed in colon cancer cells and tissues, and the high expression of EGFL7 is related to the survival time and poor prognosis of patients.

### 3.3. Silencing EGFL7 Can Inhibit the Proliferation, Invasion, and Clone Formation of Colon Cancer Cells

**Figure 3:**
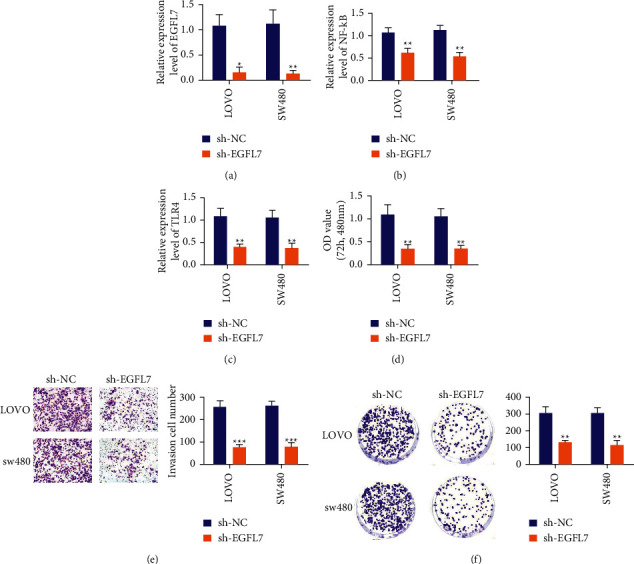
Silencing EGFL7 can inhibit the proliferation, invasion, and clone formation of colon cancer cells. (a) RT-qPCR experiment was used to test the expression of EGFL7 in LOVO and SW480 cells. (b, c) RT-qPCR experiment was used to test the level of NF-*κ*B and TLR4 after EGFL7 silencing in LOVO and SW480 cells. (d) CCK-8 experiment was used to detect cell proliferation. (e) Transwell experiments was used to detect cell invasion. (f) Cell cloning assay was used to detect clone formation of LOVO and SW480 cells. ^*∗*^*p* < 0.05, ^*∗∗*^*p* < 0.01, and ^*∗∗∗*^*p* < 0.001.

We further studied the effects of EGFL7 on the proliferation, migration, invasion, and clone formation of colon cancer cells LOVO and SW480 by silencing EGFL7. RT-qPCR experiment verified that the expression of EGFL7 in LOVO and SW480 cells in the sh-EGFL7 group was very low compared with the sh-NC group, indicating that the EGFL7 silencing in LOVO and SW480 cells was successful (Figure 3(a)). The results of the RT-qPCR experiment further showed that, after EGFL7 silencing in LOVO and SW480 cells, the expression of NF-*κ*B and TLR4 decreased significantly (Figures 3(b) and 3(c)). In addition, the CCK-8 experiment showed that EGFL7 silencing would significantly inhibit cell proliferation (Figure 3(d)). Transwell experiments showed that EGFL7 silencing would significantly inhibit cell invasion (Figure 3(e)). The results of the cell cloning assay showed that EGFL7 silencing would significantly inhibit the clone formation of LOVO and SW480 cells (Figure 3(f)). The above experimental results show that EGFL7 silencing can inhibit the proliferation, invasion, and clone formation of colon cancer cells to inhibit their malignant progress.

### 3.4. Paeoniflorin Can Alleviate Ulcerative Colitis-Associated Colon Cancer by Inhibiting the Expression of EGFL7

**Figure 4:**
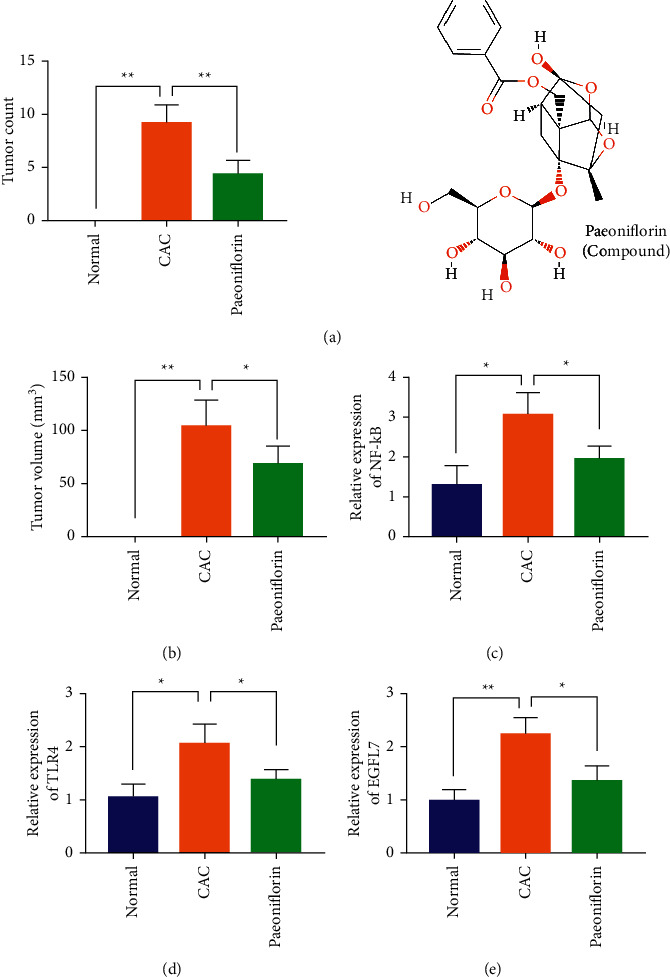
Paeoniflorin can alleviate ulcerative colitis-associated colon cancer by inhibiting the expression of EGFL7. (a, b) The number and volume of tumors in colon tissue was tested. (c–e) RT-qPCR experiment was used to detect the expression of NF-*κ*B, TLR4, and EGFL7 in colon tissue. ^*∗*^*p* < 0.05 and ^*∗∗*^*p* < 0.01.

We studied the effects of paeoniflorin on colon tissue injury, the number and volume of colon tumors in CAC model mice. Compared with the model group, after administration of paeoniflorin, the number and volume of tumors in colon tissue were significantly reduced (Figures 4(a) and 4(b)). In addition, the RT-qPCR experiment showed that paeoniflorin could effectively reduce the expression of NF-*κ*B, TLR4, and EGFL7 compared with the CAC model group. The above experiments show that paeoniflorin may effectively reduce the expression of NF-*κ*B, TLR4, and EGFL7 and further alleviate the injury of colon tissue caused by CAC and the progress of colon tumor.

### 3.5. Paeoniflorin Can Inhibit the Expression of Inflammatory Factors in Ulcerative Colitis-Related Colon Cancer

**Figure 5:**
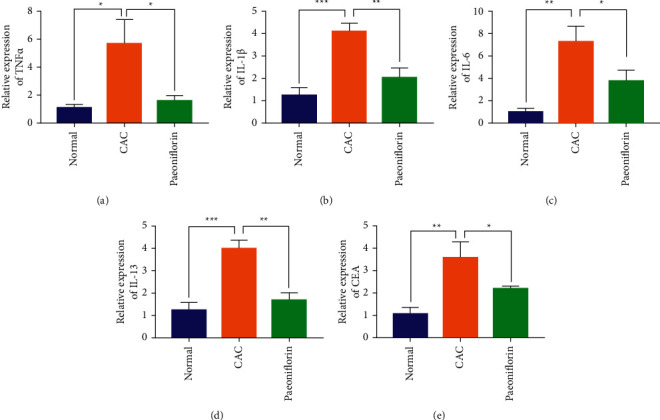
Paeoniflorin can inhibit the expression of inflammatory factors in ulcerative colitis-related colon cancer. (a–e) RT-qPCR experiment was used to detect the expression of TNF-*α*, IL-1*β*, IL-6, IL-13, and CEA. ^*∗*^*p* < 0.05, ^*∗∗*^*p* < 0.01, and ^*∗∗∗*^*p* < 0.001.

We performed RT-qPCR experiments to compare the effects of paeonionside on the expression of TNF-*α*, IL-1*β*, IL-6, IL-13, and CEA-related inflammatory factors in the colon tissues of CAC mice. The results showed that the expression of inflammatory factors was significantly reduced in the colon tissue of the paeoniflorin group compared to the model group, indicating that paeoniflorin was able to relieve CAC inflammation (Figures 5(a)–5(e)).

### 3.6. Paeoniflorin Can Inhibit the Malignant Progress of Colon Cancer Cells by Targeting EGFL7

**Figure 6:**
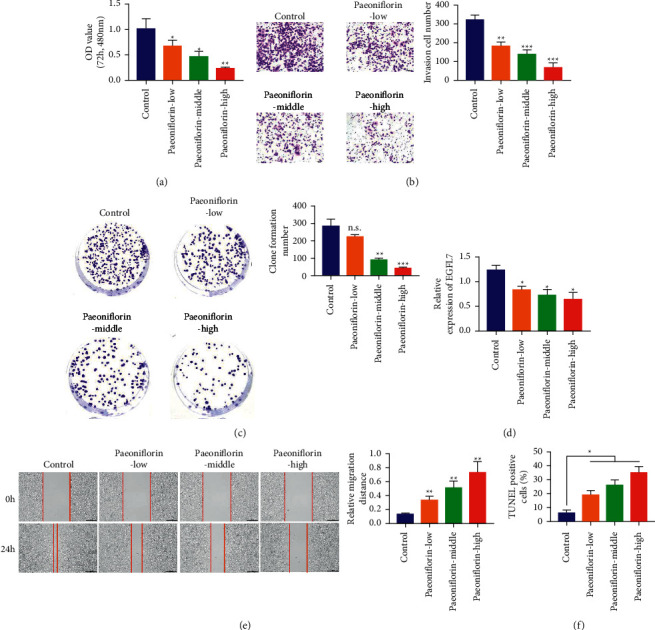
Paeoniflorin can inhibit the malignant progress of colon cancer cells by targeting EGFL7. (a) CCK-8 experiment was used to detect the effect of different concentrations of paeoniflorin on cell proliferation. (b) Transwell experiments was used to detect the effect of different concentrations of paeoniflorin on cell invasion. (c) Cell cloning assay was used to detect the effect of different concentrations of paeoniflorin on clone formation of LOVO and SW480 cells. (d) RT-qPCR was used to test the expression of EGFL7 mRNA in SW480 cells decreased after adding PF. (e) Scratch test was used to detect the effect of different concentrations of paeoniflorin on cell migration of LOVO and SW480 cells. (f) TUNEL experiments was used to detect the effect of different concentrations of paeoniflorin on apoptosis of LOVO and SW480 cells. ^*∗*^*p* < 0.05, ^*∗∗*^*p* < 0.01, and ^*∗∗∗*^*p* < 0.001.

We further validated the effects of different concentrations of paeoniflorin on proliferation, migration, invasion, clone formation, and apoptosis in colon cancer SW480 cells. SW480 cells were divided into four groups: the control group, PF-L (40 *μ*g/ml), PF-M (80 *μ*g/ml), and PF-H (160 *μ*g/ml). The experimental results of CCK-8 showed that compared with the control group, the proliferation of SW480 cells in the PF group decreased significantly, and the proliferation of SW480 cells decreased with the increase of PF concentration (Figure 6(a)). The results of the transwell experiment showed that compared with the control group, the number of SW480 cells passing through matrix gel decreased significantly, and the higher the concentration of PF, the fewer the number of SW480 cells passing through matrix gel (Figure 6(b)). The results of the clone formation experiment showed that the number of SW480 cells clones in the control group was the most, the number of cell clones decreased after PF was added, and the number of cell clones in the PF-H group was the least(Figure 6(c)). We detected the expression of EGFL7 mRNA in different groups of colon cancer SW480 cells by RT-qPCR. The results showed that compared with the control group, the expression of EGFL7 mRNA in SW480 cells decreased after adding PF and the lower expression of EGFL7 mRNA with the increase of PF concentration (Figure 6(d)). In addition, the results of the scratch and TUNEL experiments showed that PF could inhibit SW480 cell migration and promote the apoptosis of SW480 cells, respectively, which was positively correlated with the concentration of PF (Figures 6(e) and 6(f)). The above experimental results show that PF may further inhibit the proliferation, migration, invasion, and clone formation of SW480 cells and promote the apoptosis of SW480 cells by inhibiting the expression of EGFL7.

## 4. Discussion

Colorectal cancer is a common and high mortality malignancy worldwide, which seriously affects human life and health [10]. However, the current drugs have more or less obvious side effects, so we need to develop a drug with good therapeutic effects and less side effects. Recent studies have shown that PF has an anti-inflammatory effect and can alleviate rheumatoid arthritis in a rat model through anti-inflammatory effect [11,12]. Moreover, PF can inhibit lipopolysaccharide-induced inflammation by regulating the RhoA/NLRP3 signaling pathway and preventing intestinal barrier disruption [13,14]. PF can also show an inhibitory effect on tumor cell migration and invasion. UC and CAC are closely related to the inflammatory response, so we further investigated the therapeutic effect of PF on CAC. We established a CAC mouse model and colon cancer cells to study the therapeutic effect of PF on CAC mice and the inhibition of malignant progress of colon cancer cells in vivo and in vitro. The results showed that, in AOM/DSS-induced CAC mouse model, PF could significantly inhibit inflammatory factors in mouse colon tissue, protect intestinal mucosal barrier, and effectively inhibit the growth of colon tumors. In vitro, PF can significantly inhibit the proliferation, migration, invasion, and clone formation of colon cancer cells and promote cell apoptosis. Our study confirms that PF has a certain therapeutic effect and potential on CAC.

Recent studies have shown that inflammatory factors and TLR4/NF-*κ*B-mediated signaling pathways play a very important role in CAC [15–17]. TLR4/NF-*κ*B signaling pathway is closely related to the inflammatory response. TLR4 plays an important role in the recognition of pathogenic microorganisms and the control of acquired immune responses in natural immunity. After the activation of TLR4, adaptor protein MyD88 conducts signal transduction, promoting the production of some cytokines and aggravating intestinal inflammation. Studies confirmed a small amount of inflammatory infiltration in the lamina propria of the colon in TLR4 gene-deficient mice. Mice that did not have the TLR4 gene knocked out had severe intestinal inflammation. The NF-*κ*B inflammatory pathway is activated when the cytoplasmic TLR4 domain binds to the carboxyl terminus of MyD88, resulting in the massive recruitment of downstream proteins in MyD88. Activation of NF-*κ*B is a key step in the activation and proliferation of inflammatory responses in enteritis. In addition, a large number of clinical studies have shown that NF-*κ*B P65 oligonucleotides can significantly reduce the expression of NF-*κ*Bp65 and cytokines in the mucous membrane and improve the severity of clinical symptoms. Therefore, TLR4 and NF-*κ*B play a key role in the development of inflammation.

EGFL7 is a secretory protein in the epidermal growth factor-like protein family. It is secreted by endothelial cells and plays an important role in maintaining the integrity of vascular endothelial cells and promoting their adhesion and migration [18,19]. At present, the research on EGFL7 mostly focuses on angiogenesis and cancer cell invasion and metastasis, and there is no research on EGFL7 in colon cancer [20]. EGFL7 protein is an endothelial cell-specific secretory factor. It is an essential factor for vascular lumen formation. Its lack will lead to obstruction of lumen formation, thus affecting the perfection of blood vessel function. It is highly expressed in tumor and inflammatory tissues. EGFL7 is expressed in a variety of tumors, including liver cancer, malignant glioma, breast cancer, lung cancer, and pancreatic cancer. EGFL7 may be involved in the following two aspects of the process of tumor metastasis: (1) EGFL7 protein stimulates the movement and migration ability of tumor cells by autocrine or paracrine and participates in the invasion and metastasis of tumor from the primary site to the surrounding areas; (2) EGFL7 protein secreted by tumor endothelial cells facilitates the infiltration of tumor cells into the bloodstream from the primary site, leading to the formation of tumor organ-specific metastases. Neovascularization plays a very important role in tumor growth and metastasis. Blocking the expression of EGFL7 in tumor neovascularization will help inhibit tumor growth and metastasis and provide a new approach for tumor treatment.

In this study, we found that EGFL7 was overexpressed in the colon tissue of the AOM/DSS-induced CAC mice. We further examined the expression of EGFL7 in the colon cancer tissues of patients, where EGFL7 is overexpressed relative to the normal colon tissue, and the results are consistent with the CAC mice. Moreover, the expression of EGFL7 is closely related to the prognosis of colon cancer patients. The survival time of colon cancer patients with overexpression of EGFL7 is significantly lower than that of colon cancer patients with low expression of EGFL7. EGFL7 is positively correlated with the malignancy of colon cancer cells, and at the cell level, we also confirmed that the expression of EGFL7 is related to the proliferation, invasion, migration, clone formation, and apoptosis of colon cancer cells. PF could effectively reduce the expression of EGFL7 in colon cancer cells, further inhibit the proliferation, invasion, migration, and clone formation, and promote the apoptosis of colon cancer cells. PF could also effectively reduce the infiltration of inflammatory cells in colon tissue of CAC mice induced by AOM/DSS, restore the disruption of the intestinal mucosal barrier, and effectively inhibit the growth of tumors in colon tissue. In addition, our study also showed that PF can effectively inhibit the TNF-*α*, IL-1*β*, IL-6, IL-13, and CEA-related inflammatory factors expression and inhibit the TLR4/NF-*κ*B signal pathway in CAC model mice. Therefore, PF showed a potential therapeutic effect on mice in the CAC model by inhibiting TLR4/NF-*κ*B mediated inflammatory response and EGFL7 expression.

## 5. Conclusions

Our study confirmed that PF can effectively inhibit the proliferation, migration, invasion, and clone formation and promote the apoptosis of colon cancer cells. Moreover, we also found that PF had a certain therapeutic effect on CAC mice induced by AOM/DSS. We further studied the mechanism of PF in the treatment of CAC. PF plays a role in the treatment of CAC by inhibiting TLR4/NF-*κ*B-mediated inflammatory response and EGFL7 expression.

## Data Availability

The data used to support the findings of this study are available from the corresponding author upon request.
